# *Morinda citrifolia* fruit extract enhances the resistance of *Penaeus vannamei* to *Vibrio parahaemolyticus* infection

**DOI:** 10.1038/s41598-024-56173-4

**Published:** 2024-03-07

**Authors:** Julia Hwei Zhong Moh, Victor Tosin Okomoda, Nurshahieda Mohamad, Khor Waiho, Shaibani Noorbaiduri, Yeong Yik Sung, Hidayah Manan, Hanafiah Fazhan, Hongyu Ma, Muyassar H. Abualreesh, Mhd Ikhwanuddin

**Affiliations:** 1 Curtin Aquaculture Research Lab, Curtin University Malaysia, CDT 250, 98009 Miri, Sarawak Malaysia; 2 Institute of Tropical Aquaculture and Fisheries, Universiti Malaysia Terengganu, 21030 Kuala Nerus, Terengganu Malaysia; 3 Department of Fisheries and Aquaculture, College of Forestry and Fisheries, Joseph Sarwuan Tarka University (Formerly Federal University of Agriculture Makurdi), P.M.B. 2373, Makurdi, Nigeria; 4 Guangdong Provincial Key Laboratory of Marine Biotechnology, Shantou University, Shantou, 515063 Guangdong China; 5 STU-UMT Joint Shellfish Research Laboratory, Shantou University, Shantou, 515063 Guangdong China; 6 Centre for Chemical Biology, Universiti Sains Malaysia, 11900 Minden, Penang Malaysia; 7 Institute of Marine Biotechnology, Universiti Malaysia Terengganu, 21030 Kuala Nerus, Terengganu Malaysia; 8 Department of Marine Biology, Faculty of Marine Sciences, King Abdulaziz University, 21589 Jeddah, Saudi Arabia; 9 Faculty of Fisheries and Marine, Campus C, Airlangga University, Mulyorejo, Surabaya 60115 Indonesia

**Keywords:** Hepatopancreas, Histopathology, Noni, Plant extract, Shrimp, Vibriosis, Biotechnology, Environmental sciences, Limnology, Endocrinology

## Abstract

*Vibrio parahaemolyticus* is a gram-negative facultative anaerobic bacterium implicated as the causative agent of several shrimp diseases. As part of the effort to provide biocontrol and cost-effective treatments, this research was designed to elucidate the effect of *Morinda citrifolia* fruit extract on the immunity of *Penaeus vannamei* postlarvae (PL) to *V. parahaemolyticus*. The methanol extract of *M. citrifolia* was vacuum evaporated, and the bioactive compounds were detected using gas chromatography‒mass spectrometry (GC‒MS). Thereafter, *P. vannamei* PL diets were supplemented with *M. citrifolia* at different concentrations (0, 10, 20, 30, 40, and 50 mg/g) and administered for 30 days before 24 h of exposure to the bacterium *V. parahaemolyticus*. A total of 45 bioactive compounds were detected in the methanol extract of *M. citrifolia,* with cyclononasiloxane and octadecamethyl being the most abundant. The survival of *P. vannamei* PLs fed the extract supplement was better than that of the control group (7.1–26.7% survival greater than that of the control group) following *V. parahaemolyticus* infection. Shrimp fed 50 mg/g *M. citrifolia* had the highest recorded survival. The activities of digestive and antioxidant enzymes as well as hepatopancreatic cells were significantly reduced, except for those of lipase and hepatopancreatic E-cells, which increased following challenge with *V. parahaemolyticus*. Histological assessment of the hepatopancreas cells revealed reduced cell degeneration following the administration of the plant extracts (expecially those fed 50 mg/g *M. citrifolia*) compared to that in the control group. Therefore, the enhanced immunity against *V. parahaemolyticus* infection in *P. vannamei* could be associated with the improved hepatopancreas health associated with *M. citrifolia* fruit extract supplementation.

## Introduction

Shrimps have been recognized as the most common seafood products in the world^1,2^. Among the shrimp species traded, *Penaeus vannamei*, often referred to as whiteleg shrimp, is popular in many parts of the world^3,4^. The global popularity of whiteleg shrimp compared to that of other shrimp species is predicated on its fast-growing potential and tolerance to extreme environmental conditions^5^. Additionally, penaeid shrimp are far superior to other shrimp species in yield and fillet quality because they are rich in fatty acids and proteins^6,7^. These advantages may spur the expansion of *P. vannamei* culture, making P. vannamei one of the most important fisheries resources and economic activities worldwide^8,9^. However, the growth of these industries is threatened by the spread of disease.

This is because of the intensive culture of shrimp, which provides favorable conditions for disease infection. Consequently, these infections have elevated mortalities, amounting to billions of dollars in financial losses^8,10^. One such disease of concern for most shrimp farmers worldwide is acute hepatopancreatic necrosis disease (AHPND)^11–13^. It is caused by PirABVP-producing strain of *Vibrio parahaemolyticus* (VP_AHPND_) and was first observed in China^14–16^. However, losses due to AHPND disease have been reported in Southeast Asia, Mexico, and South America^17^. It is known to affect *P. vannamei* postlarvae within the first few days of stocking and often results in total mortality of the infected stocks^17,18^.

Several methods have been developed to control the spread of diseases in shrimp. Although vaccination and antibiotics are effective strategies^18–22^, the emergence of drug-resistant pathogens and the health risks associated with chemical treatments have led to the search for alternative methods^8–26^. Disease prevention through biocontrol measures has gained relevance in recent years. Research on the potential of plant parts and their extracts as important alternatives to antibiotics and chemical treatments is gaining attention^27,28^. This is due to their possession of natural resources, which have immunostimulant effects and hence can improve the immunity of the organism in which they are administered^29–31^. This treatment option is also considered for its cost-effectiveness and ability to protect the environment from chemical toxicity^32^.

The noni (*Morinda citrifolia*) is a polynesian plant found in many parts of the world and is traditionally used for the treatment of several human illnesses, such as arthritis, heart disease, diabetes, hypertension, fever, indigestions, and headaches^33–36^. Several studies have demonstrated the antimicrobial activity of Noni plants and their ability to improve the immune responses of aquaculture species^37–39^. Specifically, Rodriguez et al.^40^ reported that aqueous extracts of *M. citrifolia* improved the health of *P. vannamei* against pathogenic *Vibrio* spp. due to their antioxidant, immunomodulatory, and antimicrobial properties. Similarly, Chang et al.^41^ performed an in vitro test and showed that traditional water decocting extraction of *M. citrifolia* fruit had a bacteriostatic effect on *V. parahaemolyticus* isolated from *P. vannamei*. The improved immunity of aquatic species resulting from feed supplementation is often associated with improved activities in the digestive system^32,42–45^ and antioxidant responses^46,47^. In our earlier study, increasing concentrations of the methanolic fruit extract of *M. citrifolia* were administered to *P. vannamei* and found to improve the growth and physiological responses of the shrimps^48^. This study, therefore, was designed to elucidate the effect of the extract on the immunity of shrimp against *V. parahaemolyticus* infection. Hence, the survival, digestive enzyme activities, antioxidant responses, and histopathology of the hepatopancreas (tissue structure and hepatopancreatic cell number) were assessed before and after infection in this study.

## Methodology

### Preparation of the *Morinda citrifolia* fruit crude extract

Approximately five kilograms of *M. citrifolia* fruits obtained from the environs of Kuala Terengganu were rinsed with distilled water and subjected to extraction. In a preliminary study done (Not reported), methanol extraction of the *M. citrifolia* proved to yield more that alternative solvents such as aqueous and ethanol, hence was selected for this study. Methanol extraction of the fruits collected was performed according to the procedure described in the study by Natheer et al.^49^ with some modifications. In brief, the fruits were sliced into pieces (1 mm length × 1 mm width × 5 mm thickness) and dried in an oven (50 °C) for approximately five hours. Oven-dried samples were placed in a dry food processor and ground into powder form, followed by extraction with 70% methanol (at a ratio of 1:5, i.e., sample to methanol) for 48 h with constant stirring. The mixture obtained was then filtered through Whatman No. 1 filter paper with a diameter of 45 mm, a thickness of 180 µm, and a pore size of 11 µm. The filtrate was dried using rotary evaporation (Buchi R-215 Rotavapor System) under reduced pressure to expel the methanol. The extracts were prepared in three batches since each batch of extract prepared was sufficient for only 10 days of the feeding trial. To ensure that the extraction content was consistent in each of the batches used, the *M. citrifolia* fruits were collected from the same source, and the weight of the fruit before extraction and the percentage of water loss after drying were similar for the three batches of extraction. Additionally, the same ratio of 1:5 of the ground sample to be extracted in methanol (70%) was maintained for each batch of extractions. The final weight of the extract after the elimination of methanol was also approximately the same for all three extraction batches. Similarly, the extract and the prepared diet were kept in a chiller at 4 °C to ensure freshness and reduce degradation of the phytochemicals in the extracts during the ten days of use. It should be noted that all methods used in this study involving plants and the care or use of animals followed institutional, national, and international guidelines and legislation. Additionally, all necessary permissions and licenses needed for the collection and processing of the *M. citrifolia* fruits were obtained.

### Bioactive compound analysis with gas chromatography‒mass spectrometry (GC‒MS)

The crude extract of *M. citrifolia* fruit was subjected to GC‒MS analysis [Agilent 6890 Series GC System with 5973 Network Mass Selective Detector equipped with SGE BPX5 column (30 m in length × 250 µm in diameter × 0.25 µm in thickness)] in triplicates. Helium gas was used as the carrier gas, and the split mode was selected. The details on the back inlet were as follows:

**Table:** 

Mode	Split
Initial Temperature	280 °C
Pressure	12.04 psi
Split ratio	20:1
Split flow	20.0 ml/min

The extract sample was diluted to a 10 × dilution in methanol and injected at a flow rate of 1.0 ml/min. The injection volume was 0.2 µl. The temperature was initially set at 50 °C for 2 min. The oven temperature was then increased to 300 °C at a rate following a total run time of 49 min:

**Table:** 

Steps	Rate	Final temperature
1	5 °C/min	250 °C
2	10 °C/min	300 °C
3	0 °C/min (off)	

The GC‒MS results of the bioactive compounds detected through comparison of the retention times of standards. The mass spectra obtained were matched with the spectra available in the National Institute Standard and Technology (NIST) database (Reference Library—NIST05a.L.). The presence of the bioactive compounds detected in the extract of *M. citrifolia* fruit was expressed as a percentage based on the peak area produced in the chromatogram of the GC‒MS analysis.

### Collection and acclimatization of postlarval shrimp

The *P. vannamei* used for this study was PL 18 (mean weight = 0.06 ± 0.02 g), which was obtained from the local shrimp hatchery, iSharp, Blue Archipelago, and Terengganu branches. Before the beginning of the experiment, the samples were first acclimatized for 2 weeks in fiberglass tanks with seawater at a temperature of 28 °C and salinity of 30 ppt. The PLs were fed twice daily (at 8:00 am and 8:00 pm) during the acclimatization period with a formulated diet (brand: gold coin, 903S) containing 40% protein, 3% fiber, and 7% fat. Thereafter, the average weight and length of the shrimp at the start of the experiment were recorded as approximately 0.0816 g ± 0.01 and 22.45 mm ± 0.4, respectively.

### Feeding trial of shrimp supplemented with fruit extract.

An experimental feeding trial of *P. vannamei* PL was conducted using the protocol previously adopted by Moh et al.^48^. In brief, the PLs were randomly divided into six separate treatment tanks according to the concentration of supplemented *M. citrifolia* fruit extract. *M. citrifolia* fruit extract was supplemented with commercial shrimp feed at 0, 10, 20, 30, 40, and 50 mg/g, represented as T0, T1, T2, T3, T4 and T5, respectively. The choice of the six experimental doses was based on the findings of the range finding test previously performed before the current experiment. The shrimp that received 0 mg/g PL were used as the control group. The extract was weighed according to the concentration for each supplementation and was dissolved in sterile distilled water before it was mixed evenly with the pellets. The pellets that had been supplemented with the extract were oven-dried at 40 °C like it was reiterated earlier, and the diet preparation was performed in three batches. Each batch was kept for a maximum of 10 days in a chiller (at 4 °C) to ensure freshness and to prevent the degradation of the phytochemicals mixed with the feed. The analysis of each feeding group was performed in triplicate. Each tank (dimensions: 3.300 m × 1.150 m × 0.750 m) was filled with approximately 1200 L of treated seawater and 300 shrimp. *P. vannamei* PLs were fed a supplemented diet four times daily at regular time intervals (0800 h, 1200 h, 1600 h, and 2000 h). The feeding trial lasted for 30 days, during which the DO concentration (5.4 mg/L ± 0.041), water temperature (28 °C ± 0.023), and salinity (30 ppt ± 0.042) were maintained at their optimum levels through continuous aeration and 20% water change at 2-day intervals.

### Experimental infection with *V. parahaemolyticus*

After the 30-day feeding period, *P. vannamei* PLs were subjected to a challenge test using the bacterium *V. parahaemolyticus* through water immersion^50^. To this end, 15 shrimps weighing between 1.2 and 1.7 g from each treatment and replicate tank were transferred to a representative 1 L of treated seawater in a clear aquarium (dimensions: 18 cm × 11 cm × 9.5 cm). The challenge was performed in triplicate for each treatment tank. The AHPND strain causing *V. parahaemolyticus* was obtained from the Institute of Marine Biotechnology (IMB), Universiti Malaysia Terengganu. The PLs were challenged with the bacterium at a concentration of 2.678 × 10^6^ cfu/ml (LC_50_) for 24 h^51^. The survival of the infected PLs was monitored for 24 h, during which the dead and unresponsive shrimp were collected at 4, 8, 10, 16, and 24 h.

### Physiological responses of *P. vannamei* PL before and after the challenge test

The hepatopancreases of the *P. vannamei* PLs in all groups (5 shrimps each) were sampled from the cephalothorax for physiological analysis before and 24 h post inoculation with the *V. parahaemolyticus* used for the challenge test. The physiological aspects examined included digestive enzyme activities, antioxidant enzyme activities, and histopathology analysis of the tissue structure as well as the hepatopancreatic cells.

### Digestive enzyme activity assays

A bioassay kit from BioVision was used for the analyses of digestive enzyme activity. The digestive enzymes selected for the analysis included amylase (K711-100), lipase (K722-100), and trypsin (K771-100). The colorimetric assays were performed following the protocol provided on the label by the manufacturer.

### Antioxidant enzyme assays

The antioxidant enzyme activity of the test organisms was determined using the protocol contained in the EnzyChrom™ bioassay kit. This approach allowed the analysis of four antioxidant enzymes, namely, (i) catalase (ECAT-100), (ii) glutathione (DIGT-250), (iii) superoxide dismutase (ESOD-100), and (iv) thiobarbituric acid (DTBA-100).

### Histopathological examination of the shrimp hepatopancreas

The histology procedure was performed following the procedure adopted by Manan et al.^52^, with modifications applied during the process. 5 shrimps each from the different treatments were collected for this analysis. The hepatopancreases were extracted and fixed in Davidson’s solution for a period of 24 h, followed by tissue processing using a tissue processor. The procedure for processing the tissue included dehydration through a series of alcohol exposures, followed by clearing in xylene solutions and, finally, impregnation in paraffin wax. The wax-embedded samples were sectioned at a thickness of 5 µm and adhered to clear glass slides. Hematoxylin and eosin (H&E) staining was performed, and the stained samples were mounted with dibutylphthalate polystyrene xylene (DPX). The permanent slides of the hepatopancreas tissues were micrographed using a Nikon Eclipse 80i microscope at magnifications of 100 × and 200 ×. The hepatopancreas tissues were observed, and changes in the hepatopancreatic cells were recorded.

### Statistical analysis

A homogeneity test of the survival and physiological response data was performed before analysis. When unequal variance (P < 0.05) was obtained, ANOVA with Welch correction was performed at a confidence level of P = 0.05. This was followed by post hoc analysis for multiple comparisons between groups. Gameshowell comparison was used when Welch ANOVA was employed, whereas if Welch ANOVA was used during the analysis of variance, Tukey’s HSD test was chosen at the confidence level P = 0.05. The results obtained after analysis are presented in graphs, tables, and figures as appropriate. All the statistical analyses were performed using Minitab 14 software.

### Ethical approval

All the applicable guidelines for the use and care of animals were established in accordance with international, national, and institutional standards.

## Results

### Gas chromatography–mass spectrometry for determination of bioactive constituents

**Figure 1:**
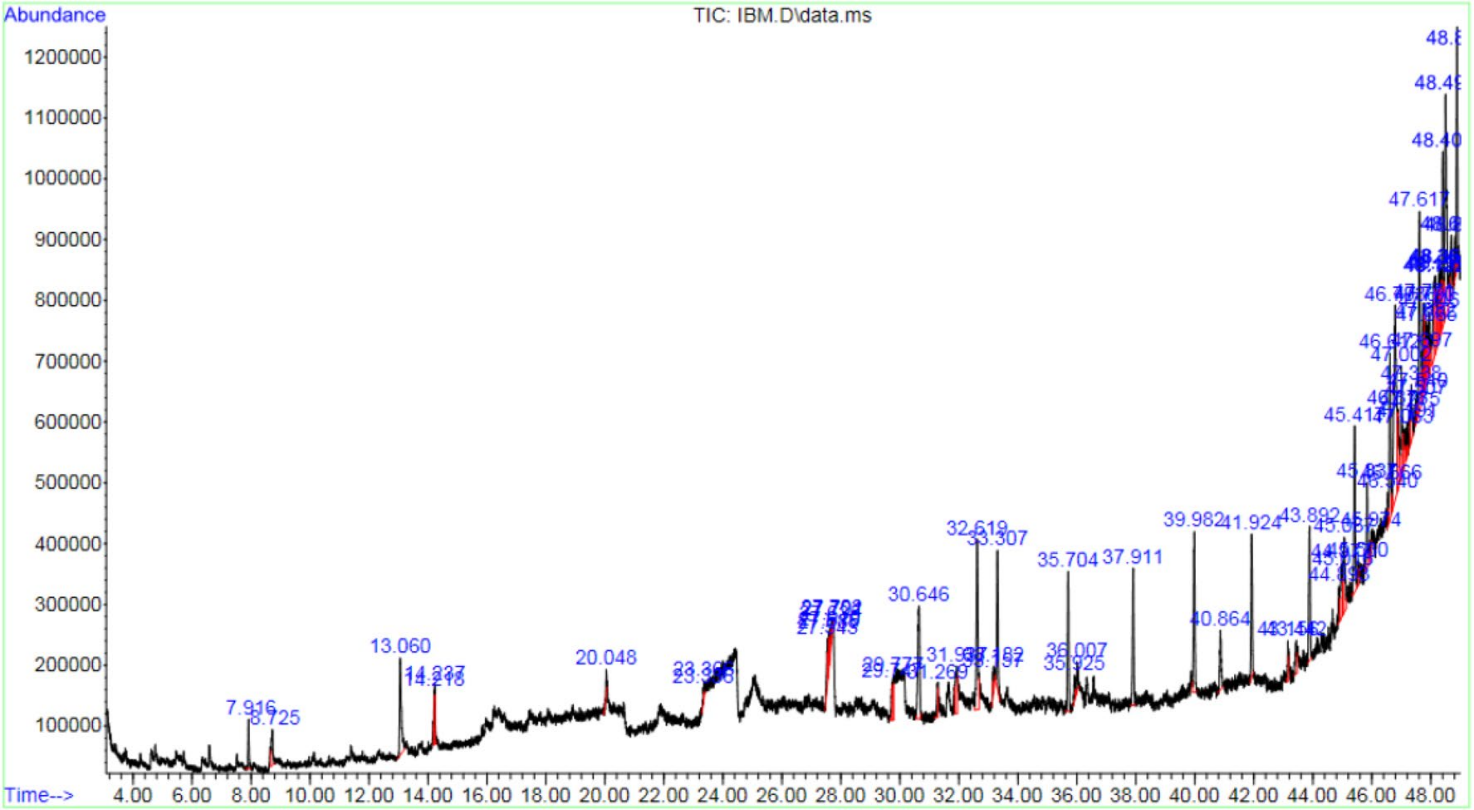
Chromatogram of the bioactive constituents detected via gas chromatography‒mass spectrometry.

A total of 45 active constituents were detected via GC‒MS analysis and are shown in Table 1. The retention times and chromatograms of the compounds detected during the analysis are presented in Table 2 and Fig. 1. Cyclononasiloxane and octadecamethyl—were found to be abundant in the fruit extract of *M. citrifolia,* covering 36.207% of the total area in the GC‒MS peak chart. 3-Amino-3-(6,7,9,10,12,13,15,16-octahydro-5,8,11,14,17-pentaoxa-benzocyclopenta-decen-2-yl)-propionic acid was the second most abundant compound and covered 7.614% of the total peak area, followed by 2-ethylacridine and n-hexadecanoic acid, which covered 4.177% and 4.038%, respectively, of the total peak area. Additional bioactive compounds detected and their abundances are listed in Table 1. 2,4-Dihydroxy-2,5-dimethyl-3(2H)-furan-3-one was detected first at a retention time of 7.91 min, followed by hexanoic acid at 8.73 min and 4H-pyran-4-one and 2,3-dihydro-3,5-dihydroxy-6-methyl- at 13.04 min. Multiple detections of the same compound at different retention times were recorded, as shown in Table 2, with some of the major compounds being octanoic acid, benzoic acid from different isomers, cyclononasiloxane, and octadecamethyl, among others.

**Table 1:** Gas chromatography‒mass spectrometry (GC‒MS) analysis of the active compounds present in the fruit extract of *M. citrifolia.*

No.	Chemical compound	Molecular formula	Molecular weight (g/mol)	Percentage area (%)
1	Cyclononasiloxane, octadecamethyl-	C_18_H_54_O_9_Si_9_	667.4	36.207
2	3-Amino-3-(6,7,9,10,12,13,15,16-octahydro-5,8,11,14,17-pentaoxa-benzocyclopenta- decen-2-yl)-propionic acid	NA	NA	7.614
3	2-Ethylacridine	C_15_H_13_N	207.27	4.177
4	n-Hexadecanoic acid (Palmitic acid)	C_16_H_32_O_2_	256.42	4.038
5	Stigmastan-3,5-diene	C_29_H_48_	396.7	3.673
6	Scillirosidin, glycoside	C_26_H_34_O_7_	458.5	2.905
7	3,4-Dihydroxymandelic acid, ethyl ester, tri-TMS	C_19_H_36_O_5_Si_3_	428.7	2.751
8	4H-Pyran-4-one, 2,3-dihydro-3,5-dihydroxy-6-methyl-	C_6_H_8_O_4_	144.12	2.619
9	Adamantane-1-(3,3-dichloropropyn-1-yl)	C_13_H_16_Cl_2_	243.17	2.577
10	Octasiloxane, 1,1,3,3,5,5,7,7,9,9,11,11,13,13,15,15-hexadecamethyl-	C_16_H_48_O_7_Si_8_	577.2	2.212
11	1H-Indole-2-carboxylic acid, 6-(4-ethoxyphenyl)-3-methyl-4-oxo-4,5,6,7-tetrahydro-, isopropyl ester	NA	NA	2.209
12	[1,1′-Biphenyl]-2,5-diol	C_12_H_10_O_2_	186.211	2.166
13	3′-Chlorooxanilic acid N'-(3-ethoxy-4-hydroxybenzylidene)hydrazide	C_17_H_16_ClN_3_O_4_	361.8	2.059
14	2-Hydroxy-3-(4-methoxy-benzyl)-succinic acid, dimethyl ester	NA	NA	1.964
15	Phenanthro[3,2-b]furan-7,11-dione, 1,2,3,4,8,9-hexahydro-4,4,8-trimethyl-, (+)-	NA	NA	1.963
16	1-Hexacosene	C_26_H_52_	364.7	1.893
17	1-cis-Styryl-4-trans-styrylbenzene	C_22_H_18_	282.378	1.412
18	2-(Acetoxymethyl)-3-(methoxycarbonyl)biphenylene	C_17_H_14_O_4_	282.29	1.383
19	Benzoic acid, 2-[(trimethylsilyl)amino]-, trimethylsilyl ester	C_13_H_22_O_3_Si_2_	282.48	1.307
20	Benz[e]azulene-3,8-dione, 5-[(acetyloxy)methyl]-3a,4,6a,7,9,10,10a,10b-octahydro-3a,10a-dihydroxy-2,10-dimethyl-, (3a.alpha.,6a.alpha.,10.beta.,10a.beta.,10b.beta.)-(+)-	C_19_H_24_O_6_	384.4	1.280
21	1H-Pyrido[3,4-b]indole, 2,3,4,9-tetrahydro-1-methyl-	NA	NA	1.241
22	Octanoic acid	C_8_H_16_O_2_	144.21	1.187
23	Dihydrotachysterol	C_28_H_46_O	398.7	0.881
24	Hexanoic acid	C_6_H_12_O_2_	116.16	0.859
25	Gibb-3-ene-1,10-dicarboxylic acid, 2,4a-dihydroxy-1-methyl-8-methylene-, 1,4a-lactone, 10-methyl ester, (1.alpha.,2.beta.,4a.alpha.,4b.beta.,10.beta.)-	NA	NA	0.712
26	1-Naphthol, 2,5,8-trimethyl-	C_13_H_14_O	186.25	0.706
27	Bicyclo[4.1.0]hept-4-en-2,3-dicarboxylic acid, 5,6,7,7-tetramethyl-, dimethyl ester	NA	NA	0.690
28	2,4-Dihydroxy-2,5-dimethyl-3(2H)-furan-3-one	C_6_H_8_O_4_	144.12	0.689
29	4H-1-Benzopyran-4-one, 5-hydroxy-7-methoxy-2-(4-methoxyphenyl)-	NA	NA	0.648
30	Silicic acid, diethyl bis(trimethylsilyl) ester	C_10_H_28_O_4_Si_3_	296.58	0.593
31	2-Hydroxy-4-isopropylnaphthalene	C_13_H_14_O	186.25	0.586
32	Z-5-Methyl-6-tetradecen-1-ol acetate	C_17_H_32_O_2_	268.435	0.571
33	4-Dehydroxy-N-(4,5-methylenedioxy-2-nitrobenzylidene)tyramine	C_16_H_14_N_2_O_4_	298.29	0.542
34	cis-2,3-Di-p-tolyl-1-phthalimido-azimine	NA	NA	0.523
35	5-Bromo-n-pentanol, cyclohexyl ether	C_11_H_21_BrO	249.19	0.483
36	Androst-5,7,9(11)-triene, 3-acetoxy-17-oxo-	NA	NA	0.424
37	Benzo[h]quinoline, 2,4-dimethyl-	C_15_H_13_N	207.27	0.411
38	Butanedioic acid, 2,3-diphenyl-, dimethyl ester	C_18_H_18_O_4_	298.3	0.385
39	1H-2-Indenone,2,4,5,6,7,7a-hexahydro-3-(1-methylethyl)-7a-methyl	C_13_H_20_O	192.3	0.340
40	1,3-Benzenedithiol	C_6_H_6_S_2_	142.2	0.257
41	trans-4-Ethoxy-4′-methoxychalcone	C_18_H_18_O_3_	282.3	0.250
42	7-Chloro-2-cyclohexyl-4[3H]quinazolinone	NA	NA	0.223
43	7-Hydroxy-6-methoxy-2H-1-benzopyran-2-one	NA	NA	0.178
44	2,4(1H,3H)-Quinolinedione, 3-benzoyl-3-(phenylmethyl)-	C_23_H_17_NO_3_	355.4	0.124
45	Benzoic acid, 2,4-bis[(trimethylsilyl)oxy]-, trimethylsilyl ester	C_16_H_30_O_4_Si_3_	370.66	0.090

**Table 2:** Retention time (in minutes) of bioactive compounds detected during GC‒MS analysis.

Retention time (min)	Bioactive compounds
7.91	2,4-Dihydroxy-2,5-dimethyl-3(2H)-furan-3-one
8.73	Hexanoic acid
13.06	4H-Pyran-4-one,2,3-dihydro-3,5-dihydroxy-6-methyl-
14.22	Octanoic acid
14.24	Octanoic acid
20.05	1,3-Benzenedithiol
23.34	2,4(1H,3H)-Quinolinedione,3-benzoyl-3-(phenylmethyl)-
23.37	Benzoic acid, 2,4-bis[(trimethylsilyl)oxy]-, trimethylsilyl ester
27.54	Cyclononasiloxane, octadecamethyl-
27.58	Cyclononasiloxane, octadecamethyl-
27.61	Cyclononasiloxane, octadecamethyl-
27.64	Cyclononasiloxane, octadecamethyl-
27.70	Cyclononasiloxane, octadecamethyl-
27.72	Cyclononasiloxane, octadecamethyl-
29.75	1-Naphthol, 2,5,8-trimethyl-
29.78	2-Hydroxy-4-isopropylnaphthalene
30.64	Cyclononasiloxane, octadecamethyl-
31.27	cis-2,3-Di-p-tolyl-1-phthalimido-azimine
31.94	1H-Pyrido[3,4-b]indole, 2,3,4,9-tetrahydro-1-methyl-
32.62	n-Hexadecanoic acid
33.16	7-Hydroxy-6-methoxy-2H-1-benzopyran-2-one
33.18	1H-2-Indenone,2,4,5,6,7,7a-hexahydro-3-(1-methylethyl)-7a-methyl
33.31	3,4-Dihydroxymandelic acid, ethyl ester, tri-TMS
35.70	Cyclononasiloxane, octadecamethyl-
35.92	5-Bromo-n-pentanol, cyclohexyl ether
36.01	Z-5-Methyl-6-tetradecen-1-ol acetate
37.91	Cyclononasiloxane, octadecamethyl-
39.98	Cyclononasiloxane, octadecamethyl-
40.87	Benzoic acid, 2-[(trimethylsilyl)amino]-, trimethylsilyl ester
41.92	Cyclononasiloxane, octadecamethyl-
43.16	1-Hexacosene
43.44	Bicyclo[4.1.0]hept-4-en-2,3-dicarboxylic acid, 5,6,7,7-tetramethyl-, dimethyl ester
43.89	Cyclononasiloxane, octadecamethyl-
44.89	4H-1-Benzopyran-4-one, 5-hydroxy-7-methoxy-2-(4-methoxyphenyl)-
44.98	1-cis-Styryl-4-trans-styrylbenzene
45.02	trans-4-Ethoxy-4′-methoxychalcone
45.06	2-Hydroxy-3-(4-methoxy-benzyl)-succinic acid, dimethyl ester
45.42	Cyclononasiloxane, octadecamethyl-
45.53	Butanedioic acid, 2,3-diphenyl-, dimethyl ester
45.84	1-Hexacosene
45.97	Androst-5,7,9(11)-triene, 3-acetoxy-17-oxo-
46.54	Octasiloxane, 1,1,3,3,5,5,7,7,9,9,11,11,13,13,15,15-hexadecamethyl-
46.61	Cyclononasiloxane, octadecamethyl-
46.67	7-Chloro-2-cyclohexyl-4[3H]quinazolinone
46.79	3-Amino-3-(6,7,9,10,12,13,15,16-octahydro-5,8,11,14,17-pentaoxa-benzocyclopenta- decen-2-yl)-propionic acid
46.88	Phenanthro[3,2-b]furan-7,11-dione, 1,2,3,4,8,9-hexahydro-4,4,8-trimethyl-, (+)-
47.00	[1,1′-Biphenyl]-2,5-diol
47.06	Dihydrotachysterol
47.19	4-Dehydroxy-N-(4,5-methylenedioxy-2-nitrobenzylidene)tyramine
47.28	Octasiloxane, 1,1,3,3,5,5,7,7,9,9,11,11,13,13,15,15-hexadecamethyl-
47.34	Adamantane-1-(3,3-dichloropropyn-1-yl)
47.51	Octasiloxane, 1,1,3,3,5,5,7,7,9,9,11,11,13,13,15,15-hexadecamethyl-
47.54	2-(Acetoxymethyl)-3-(methoxycarbonyl)biphenylene
47.62	Cyclononasiloxane, octadecamethyl-
47.70	1H-Indole-2-carboxylic acid, 6-(4-ethoxyphenyl)-3-methyl-4-oxo-4,5,6,7-tetrahydro-, isopropyl ester
47.77	3′-Chlorooxanilic acid N'-(3-ethoxy-4-hydroxybenzylidene)hydrazide
47.79	2-Ethylacridine
47.83	Octasiloxane, 1,1,3,3,5,5,7,7,9,9,11,11,13,13,15,15-hexadecamethyl-
47.86	Gibb-3-ene-1,10-dicarboxylic acid, 2,4a-dihydroxy-1-methyl-8-methylene-, 1,4a-lactone, 10-methyl ester, (1.alpha.,2.beta.,4a.alpha.,4b.beta.,10.beta.)-
47.95	2-Ethylacridine
48.12	2-Ethylacridine
48.15	1H-Indole-2-carboxylic acid, 6-(4-ethoxyphenyl)-3-methyl-4-oxo-4,5,6,7-tetrahydro-, isopropyl ester
48.27	Adamantane-1-(3,3-dichloropropyn-1-yl)
48.30	Silicic acid, diethyl bis(trimethylsilyl) ester
48.36	2-(Acetoxymethyl)-3-methoxycarbonyl)biphenylene
48.40	Scillirosidin, glycoside
48.50	Cyclononasiloxane, octadecamethyl-
48.69	Benz[e]azulene-3,8-dione,5-[(acetyloxy)methyl]-3a,4,6a,7,9,10,10a,10b-octahydro-3a,10a-dihydroxy-2,10-dimethyl-, (3a.alpha.,6a.alpha.,10.beta.,10a.beta.,10b.beta.)-(+)-
48.83	Benzo[h]quinoline, 2,4-dimethyl-
48.89	Stigmastan-3,5-diene

### Survival of the shrimp during the challenge test with *V. parahaemolyticus*

**Figure 2:**
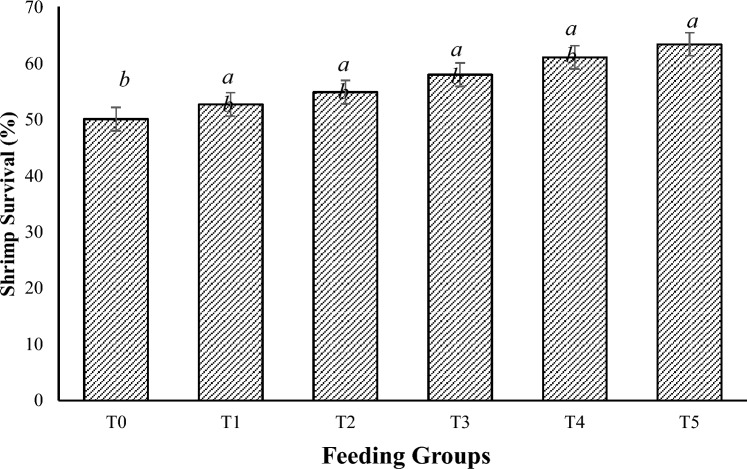
Survival of *P. vannamei* PLs from different feeding groups after 24 h of challenge with *V. parahaemolyticus*. Bars with different letters differ significantly. T0, T1, T2, T3, T4, and T5 represent 0, 10, 20, 30, 40, and 50 mg/g, respectively.

The results showed that supplementation with higher concentrations of *M. citrifolia* fruit extract increased the survival of the shrimp during *V. parahaemolyticus* challenge (P = 0.024), with the highest supplementation concentration of 50 mg/g resulting in significantly greater survival than that of the control. However, below this level of supplementation, the performance was similar to that of the control and the highest treatment tested, as shown in Fig. 2.

### Digestive enzyme activities

**Figure 3:**
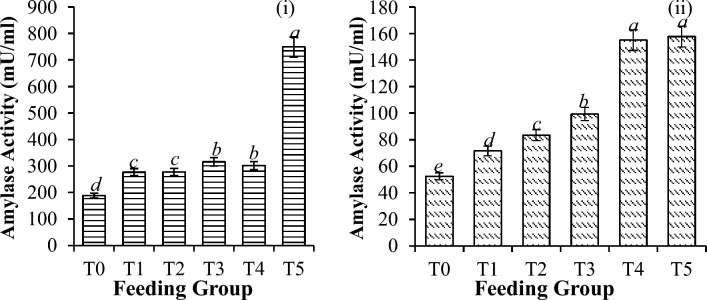
Amylase activity in *P. vannamei* PLs (**i**) before and (**ii**) after challenge with *V. parahaemolyticus*. Bars with different letters differ significantly. T0, T1, T2, T3, T4, and T5 represent 0, 10, 20, 30, 40, and 50 mg/g, respectively.

**Figure 4:**
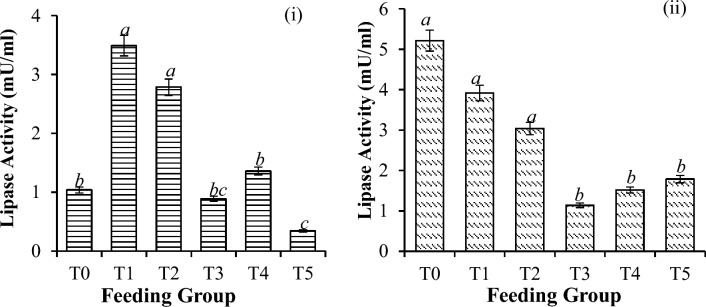
Lipase activity in *P. vannamei* PLs (**i**) before and (**ii**) after challenge with *V. parahaemolyticus*. Bars with different letters differ significantly. T0, T1, T2, T3, T4, and T5 represent 0, 10, 20, 30, 40, and 50 mg/g, respectively.

Significant differences in digestive enzyme activities were observed for all the shrimp groups. Generally, amylase activity was greater in the higher supplementation groups (10 mg/g onward) than in the control group (P < 0.001) before and after challenge with *V. parahaemolyticus* (Fig. 3). The overall activity of amylase decreased by 78.9% (P < 0.001), as shown in Table 3. However, before the challenge test, the lipase activity significantly increased in the order of 10, 20, and 40 mg/g (Fig. 4). The control had similar values to those of the 30 mg/g treatment and were greater than those obtained in the 50 mg/g treatment.

**Table 3:** Changes in digestive enzyme activity (%) in shrimp before and after challenge with *V. parahaemolyticus*.

	T0	T1	T2	T3	T4	T5
Amylase	72.19 ± 0.63^***c***^	74.13 ± 1.41^***c***^	69.94 ± 1.91^***b***^	68.56 ± 2.23^***b***^	48.69 ± 18.12^***a***^	78.95 ± 1.11^***d***^
*Lipase	401.95 ± 64.11^***e***^	13.05 ± 72.30^***a***^	24.09 ± 21.97^***b***^	30.59 ± 17.56^***c***^	51.67 ± 205.72^***d***^	423.96 ± 44.28^***e***^
Trypsin	63.70 ± 24.99^*c*^	85.98 ± 12.62^***d***^	48.38 ± 8.12^***b***^	43.32 ± 36.82^***b***^	5.89 ± 68.50^***a***^	49.87 ± 19.74^***b***^

The amount of digestive enzymes with aetheric substituents (*) increased.

Significant differences are indicated by the italicized letters, where identical letters indicate no significant difference between feeding groups. All the values are presented as the means ± SDs. T0, T1, T2, T3, T4, and T5 represent 0, 10, 20, 30, 40, and 50 mg/g, respectively.

**Figure 5:**
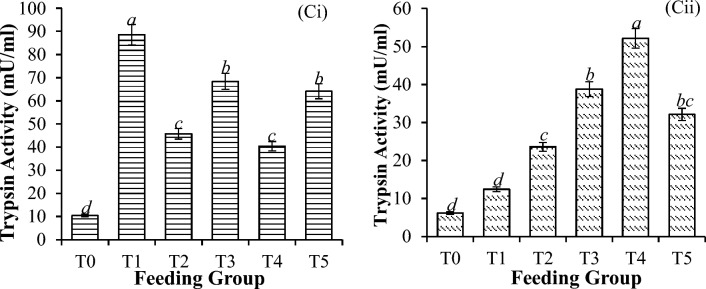
Trypsin activity in *P. vannamei* PLs (**i**) before and (**ii**) after challenge with *V. parahaemolyticus*. Bars with different letters differ significantly. T0, T1, T2, T3, T4, and T5 represent 0, 10, 20, 30, 40, and 50 mg/g, respectively.

Upon challenge with *V. parahaemolyticus,* the lipase activity significantly increased in all the treatments, reaching 423% (Table 3), with the control having the highest values recorded (Fig. 4). The trypsin activity before the challenge test was greater in the treatment group than in the control group (Fig. 5). After challenge with *V. parahaemolyticus*, there was a significant reduction in the overall activity of trypsin, reaching 86.0% (P < 0.001), as shown in Table 3. The trypsin activity was similar in the lower groups (i.e., 0 and 10 mg/g) (P = 0.451) but greater in the supplementation groups (i.e., 20 mg/g onward) (P < 0.001), as shown in Fig. 5.

### Antioxidant enzymes

**Figure 6:**
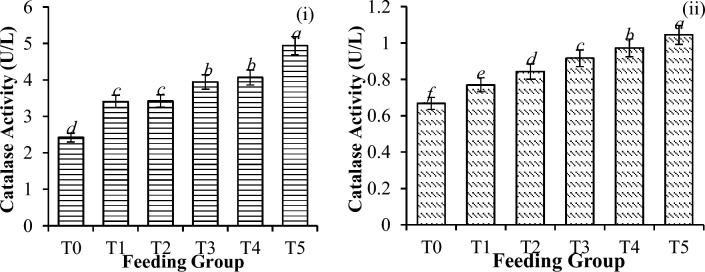
Catalase activity (U/L) in *P. vannamei* PLs (**i**) before and (**ii**) after challenge with *V. parahaemolyticus*. Bars with different letters differ significantly. T0, T1, T2, T3, T4, and T5 represent 0, 10, 20, 30, 40, and 50 mg/g, respectively.

**Figure 7:**
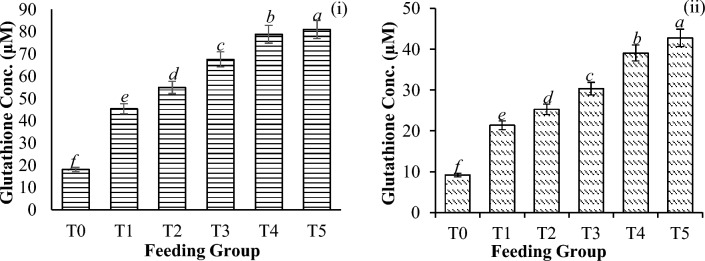
Glutathione Conc. (μM) in *P. vannamei* PLs (**i**) before and (**ii**) after challenge with *V. parahaemolyticus*. Bars with different letters differ significantly. T0, T1, T2, T3, T4, and T5 represent 0, 10, 20, 30, 40, and 50 mg/g, respectively.

**Figure 8:**
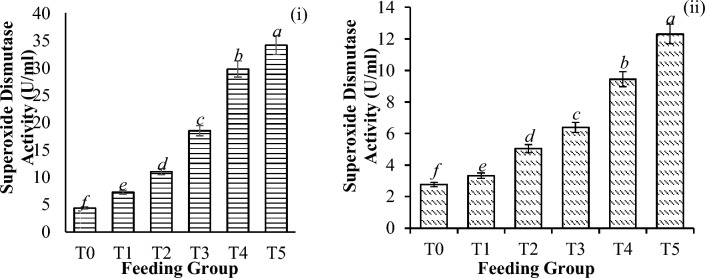
Superoxide dismutase activity (U/ml) in *P. vannamei* PLs (**i**) before and (**ii**) after the challenge test with *V. parahaemolyticus*. Bars with different letters differ significantly. T0, T1, T2, T3, T4, and T5 represent 0, 10, 20, 30, 40, and 50 mg/g, respectively.

**Figure 9:**
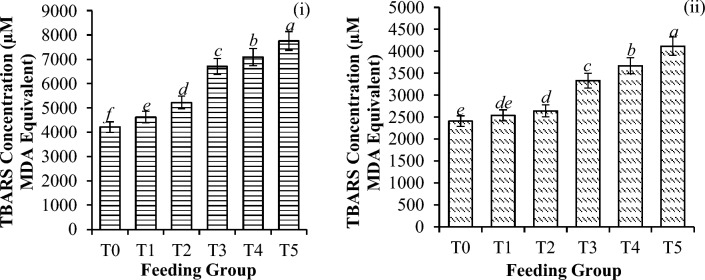
TBARS concentration (µM MDA equivalent) in *P. vannamei* PLs (**i**) before and (**ii**) after challenge with *V. parahaemolyticus*. Bars with different letters differ significantly. T0, T1, T2, T3, T4, and T5 represent 0, 10, 20, 30, 40, and 50 mg/g, respectively.

Significant differences in catalase activity (CAT) (Fig. 6), glutathione concentration (GSH) (Fig. 7), superoxide dismutase activity (SOD) (Fig. 8), and thiobarbituric acid reactive substances (TBARS) activity (Fig. 9) were detected among all the feeding groups. However, the higher supplemented groups (T3-T5) demonstrated the highest activities (CAT: P < 0.001; GSH: P < 0.001; SOD: P < 0.001; TBARS: P < 0.001), as shown before and after the challenge with *V. parahaemolyticus*.

In general, *V. parahaemolyticus* infection caused a significant reduction in the expression of all the antioxidant enzymes (CAT-78.8%, P < 0.001; GSH-55.0%, P < 0.001; SOD-68.3%, P < 0.001; and TBARS-50.4%, P < 0.001), as shown in Table 4.

**Table 4:** Changes in antioxidant enzyme activities (reduction %) of shrimp before and after the challenge test with *V. parahaemolyticus*.

	T0	T1	T2	T3	T4	T5
Catalase	72.43 ± 0.33^***c***^	77.41 ± 0.28^***ba***^	75.39 ± 0.09^***b***^	76.77 ± 0.17^***b***^	76.06 ± 0.14^***b***^	78.84 ± 0.20^***a***^
Glutathione	49.17 ± 1.00^***b***^	52.91 ± 1.98^***a***^	53.94 ± 1.38^***a***^	55.04 ± 4.85^***a***^	50.45 ± 3.97^***ba***^	47.11 ± 3.19^***c***^
SOD	36.63 ± 4.61^***c***^	54.17 ± 2.05^***b***^	54.22 ± 0.32^***b***^	65.48 ± 0.21^***a***^	68.28 ± 1.09^***d***^	63.95 ± 3.13^***a***^
TBARS	42.88 ± 1.52^***c***^	45.09 ± 0.49^***b***^	49.37 ± 3.93^***a***^	50.35 ± 1.61^***a***^	48.29 ± 0.78^***a***^	46.95 ± 1.02^***b***^

Significant differences are indicated by the italic letters. Different letters indicate significant differences between feeding groups. All the values are presented as the means ± SDs. T0, T1, T2, T3, T4, and T5 represent 0, 10, 20, 30, 40, and 50 mg/g, respectively.

### Hepatopancreas analysis

**Figure 10:**
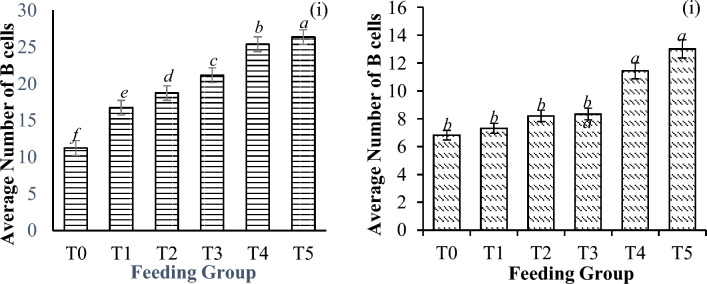
Average number of B cells present per tubule in the hepatopancreas of *P. vannamei* PLs (**i**) before and (**ii**) after challenge with *V. parahaemolyticus*. Bars with different letters differ significantly. T0, T1, T2, T3, T4, and T5 represent 0, 10, 20, 30, 40, and 50 mg/g, respectively.

**Figure 11:**
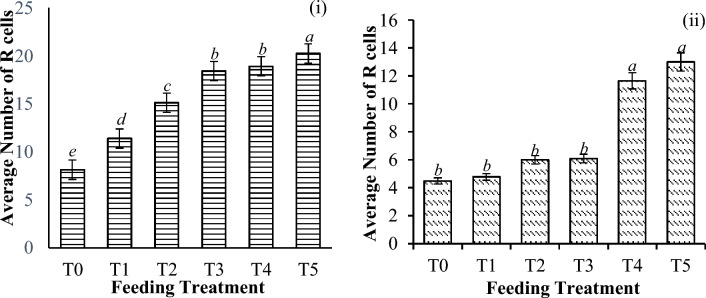
Average number of R cells present per tubule in the hepatopancreas of *P. vannamei* PL (**i**) before and (**ii**) after the challenge test with *V. parahaemolyticus*. Bars with different letters differ significantly. T0, T1, T2, T3, T4, and T5 represent 0, 10, 20, 30, 40, and 50 mg/g, respectively.

**Figure 12:**
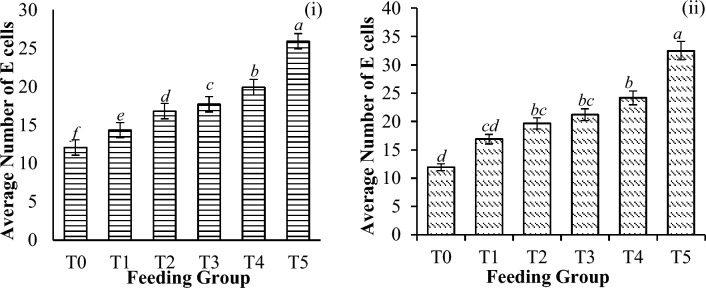
Average number of E cells present per tubule in the hepatopancreas of *P. vannamei* PLs (**i**) before and (**ii**) after challenge with *V. parahaemolyticus*. Bars with different letters differ significantly. T0, T1, T2, T3, T4, and T5 represent 0, 10, 20, 30, 40, and 50 mg/g, respectively.

From the analysis of hepatopancreatic cells (B, R, and E cells), higher cell numbers were found with increasing concentrations of *M. citrifolia*. A significant increase in these parameters was observed with increased extract supplementation before the challenge. The pattern of hepatopancreatic cells after challenge can be subdivided into two performance groups. The lower supplementation groups (0–30 mg/g) had lower cell counts than did the higher supplementation groups (40 and 50 mg/g), in which a greater number of cells (B cells: P < 0.001; R cells: P < 0.001; E cells: P < 0.001) were observed, as shown in Figs. 10, 11 and 12.

Generally, the percentages of B and R cells were significantly lower postchallenge (B cells, P = 0.002; R cells, P < 0.001) by 60.6% and 66.9%, respectively. However, compared with those observed before the challenge, the number of E cells significantly increased, and the increase was significantly different across the treatment groups (P = 0.016), as shown in Table 5.

**Table 5:** Changes in the hepatopancreatic cell (in %) activities of shrimp before and after challenge with *V. parahaemolyticus*. Hepatopancreatic cells with aetheric discordance (*).

	T0	T1	T2	T3	T4	T5
B cells (%)	39.26 ± 12.71^*d*^	56.27 ± 9.15^*b*^	56.15 ± 12.09^*b*^	60.59 ± 8.186^*a*^	54.87 ± 13.80^*b*^	50.57 ± 5.48^*c*^
R cells (%)	44.73 ± 11.66^*c*^	58.01 ± 10.80^*b*^	60.31 ± 19.05^*b*^	66.92 ± 5.40^*a*^	40.78 ± 19.89^*c*^	35.72 ± 19.06^*d*^
*E cells (%)	16.39 ± 5.87^*c*^	18.55 ± 13.87^*c*^	22.63 ± 17.56^*ab*^	23.53 ± 16.81^*ab*^	25.07 ± 20.64^*a*^	25.45 ± 21.30^*a*^

Significant differences are indicated by the italicized letters, where identical letters indicate no significant difference between feeding groups. All the values are presented as the means ± SDs. T0, T1, T2, T3, T4, and T5 represent 0, 10, 20, 30, 40, and 50 mg/g, respectively.

**Figure 13:**
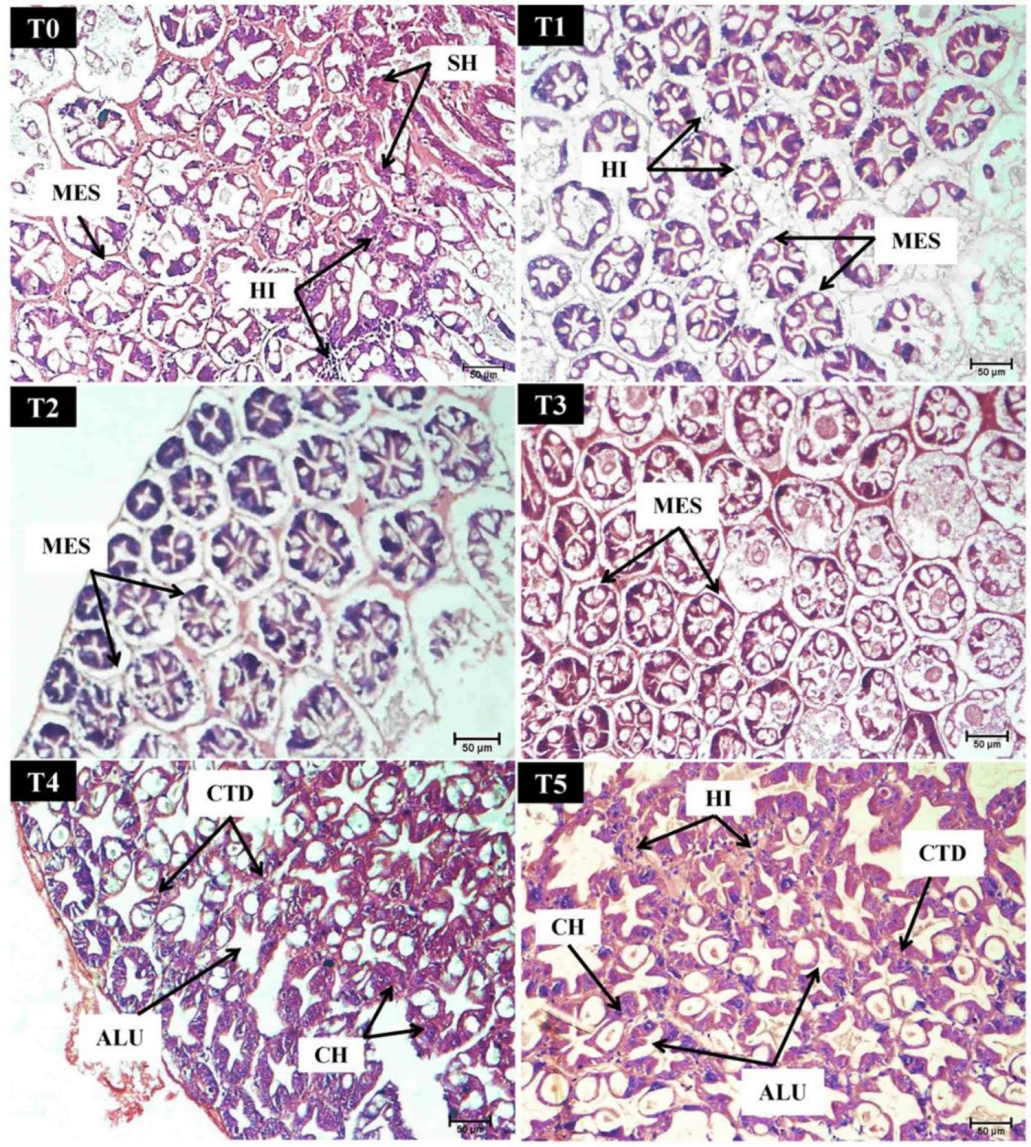
Hepatopancreatic tissue of *P. vannamei* PLs that survived the challenge test with *V. parahaemolyticus* after 24 h of supplementation with different concentrations of *M. citrifolia* fruit extract under 200 × magnification. A slight sloughing of hepatopancreatic tubules (SH) and degeneration of connective tissue (CTD) were observed in the T0, T4, and T5 treatment groups. Slight hemocytic infiltration (HI) was observed in all groups, with more HI in the T4 and T5 treatment groups. Cell hypertrophy (CH) was observed in the T4 and T5 treatment groups. B cells, R cells, and E cells in the hepatopancreatic tubules appeared to be unaffected. Separation between the myoepithelial layer and the epithelium (MES) was observed in the T0-T3 treatment groups. An abnormal lumen shape (ALU) was noticeable in the T4 and T5 feeding groups. T0, T1, T2, T3, T4, and T5 represent 0, 10, 20, 30, 40, and 50 mg/g, respectively.

The hepatopancreas tissues of *P. vannamei* PLs after the 24-h challenge test with *V. parahaemolyticus* are shown in Fig. 13. Some disintegration of the hepatopancreatic tubules and connective tissues was observed in the control (0 mg/g) and higher supplementation groups (40 and 50 mg/g). However, this histological degeneration was more severe in the former than in the latter. Hemocyte infiltration was observed in all the shrimp in all the feeding groups, especially in the 40 and 50 mg/g treatment groups. Additionally, separation of the myoepithelial layer from the epithelium was observed in the lower supplementation groups (0–30 mg/g). Notably, enlarged cells and healthy hepatopancreatic cells were observed among the severely damaged hepatopancreas tissues. Despite the structural changes in the hepatopancreas tissue of the shrimp, most of the tubular lumen still retained its distinctive star shape.

## Discussion

Disease outbreaks are the primary constraint on aquaculture expansion and productivity; hence, identifying environmentally friendly control procedures is critical for aquaculture development^51^. This study revealed 45 bioactive compounds detected in the methanol extract of *M. citrifolia* fruit, some of which have been previously reported to have medicinal properties, such as antioxidant, anticancer, and anti-inflammatory effects^48,51^. Similarly, increasing survival of the shrimp PL during *V. parahaemolyticus* challenge was observed with increasing concentrations of the plant fruit extract. This finding indicated that the improvement in the resistance of *P. vannamei* PL against *V. parahaemolyticus* was dose dependent. This finding is supported by the results of previous studies describing the potential of plant extracts as immunostimulators in shrimp. Improved survival has been demonstrated in several aquaculture species treated with Indian herbs^53^, brown seaweed, *Phaeophyceae* polysaccharide extract^54^, or green tea extract^55^ and challenged with disease-causing pathogens. Plant extracts are known to contain active compounds whose activities have been linked to enhancing innate immune responses in aquaculture species^56^. Previously, *M. citrifolia* was reported to contain high levels of phenolics and flavonoids, as well as polysaccharides and glycoproteins^48^. These compounds, which are known for their antioxidant properties^29^, could explain the improved survival of *M. citrifolia* fruit extract-fortified *P. vannamei* PLs challenged with *V. parahaemolyticus*.

Digestive system enzymes are important in immunological studies because they can impact the ability of an organism to create a barrier against pathogenic infection^57^. Zokaeifar et al.^58^ reported that improved digestive enzyme activities are often associated with improved colonization of gut bacteria and, consequently, improved efficiency of pathogen elimination by the body. The evaluation of the shrimp in this study demonstrated that amylase activity increased with increasing concentrations of the plant fruit extract. Therefore, a strong correlation between the survival of the shrimp and amylase activity was observed, indicating that a higher concentration of the plant fruit extract increased the survival of the shrimp. Trypsin is another well-known immune response trigger in all organisms^59^. These findings align with the results of Li et al.^60^, who reported improved immunity with increasing trypsin activity. The authors also explained the role of trypsin in producing hemocyanin peptide in *P. vannamei* in response to pathogen infection during a challenge test with *V. parahaemolyticus* and *Staphylococcus aureus*^60^.

The shrimp hepatopancreas is recognized as an important metabolic center for physiological balance, including enzyme production for both digestion and the immune response^61,62^. Lipase, which is stored in the hepatopancreas of shrimp, plays an important role in lipid breakdown^63^. The lower lipase activities in the shrimp with increasing concentrations of supplementation in the diet could be indicative of decreased energy needs following treatment with the extract^64^. This is justified by the fact that lipase activities significantly increased after the 24-h challenge test, a condition that would have triggered energy dispersion for osmoregulation and defense against the infection. In line with this hypothesis, in a study by Schleder et al., lipase was reported to play a significant role in improving shrimp immune responses by increasing the utilization of lipids to improve the resistance of *P. vannamei* PL to *V. parahaemolyticus* infection^65^. In contrast, amylase and trypsin activities were reduced following the challenge test. The response of amylase to stress was previously reported to be relatively sensitive, which makes it a bioindicator of stress in the environment^66^.

The shrimps also demonstrated greater antioxidant enzyme activities with increasing concentrations of the supplement. However, all the antioxidant enzymes exhibited a reduction in activity after the challenge test. The levels of antioxidant enzymes, especially SOD and CAT, are generally reduced in shrimp after pathogen infection^67,68^. GSH in *M. rosenbergii* reportedly decreased after it was challenged with *V. anguillarium*m^69^. This reduction in antioxidant activity suggested that antioxidant enzymes were utilized during the stress conditions. A reduction in SOD activity was also reported in *P. vannamei* during stressful handling conditions according to the report of Mercier et al.^70^. Compared to those in the control group in this study, the antioxidant activities in the supplementation groups were greater. Perhaps this means that the antioxidant responses of the shrimps were slower in the control group than in the treatment group^68^. The slow response may have been one of the reasons for the lower survival in the control group. Therefore, *M. citrifolia* fruit extract may enhance the immune response of *P. vannamei* PL against *V. parahaemolyticus* through the manipulation of antioxidant enzyme activities. CAT and GSH activities often overlap during oxidative stress, where GSH functions only as a cofactor during H_2_O_2_ removal^71^. This explains the lower reduction in GSH compared to that in CAT in this study.

The analysis of the hepatopancreas cells of *P. vannamei* PLs is another important index of immunity. R cells play an important role in nutrient absorption and storage as well as the excretion and detoxification of foreign substances^72^. R cells are also involved in lipid accumulation for healthier growth and stress prevention in shrimp^73,74^. Therefore, it is no surprise that the current study revealed an increase in the R cell number with the supplementation of the fruit extract. This approach may help to significantly reduce the impact of stressful shrimp exposure to *V. parahaemolyticus* infection, in line with the findings of Bhavan and Geraldine^75^. Boudet et al.^72^ also explained that cells lack apocrine secretion ability, thus making them the most vulnerable cells during stress exposure. This means that stressful circumstances cause R cells to continuously store contaminants until the cell dies from toxin poisoning^72,75,76^. This explains the significant reduction in the number of R cells after challenge with the pathogen from *V. parahaemolyticus*.

Since B cells play an important role in intracellular digestion and nutrient absorption, they are believed to encounter a similar fate as R cells, causing the cells to die from toxin accumulation. The loss of B and R cells is often augmented by mitotic cell division and the activity of E cells at the distal end of tubules^72^. This explains the increase in the number of E-cells after infection with *V. parahaemolyticus,* as observed in the present study. Therefore, the balance of cell death caused by infection, viz. a viz. cell regeneration may determine whether shrimp survive or die. Moreover, since B cells are known to be derived from F cells^77^, infection may also damage F cells, hence affecting their conversion into B cells and reducing their availability. E cells, on the other hand, are actively dividing embryonic cells involved in cell renewal in the tubular epithelium^78^, hence explaining their increase despite pathogenic infection.

Although gills are known to constitute the initial line of defense for the immunity of crustaceans against pathogens and other oxidative stresses^79^, the hepatopancreas plays an important physiological role in system optimization^75^. The function of the hepatopancreas is analogous to that of the liver in higher organisms, and the hepatopancreas is sensitive and often prone to injuries caused by infection or other stress exposure^75^. The histopathology results in this study agree with previous studies on hepatopancreatic histological degeneration following pathogenic infection^79–82^. The destruction of the hepatopancreas structure often affects its function in terms of digestion, nutrient absorption, and secretion, hence leading to the deterioration of shrimp health and ultimately mortality^72,79^. The appearance of enlarged cells in the infected hepatopancreas is also worth mentioning. Several explanations have been articulated in the past regarding the enlargement of hepatopancreatic cells. One such complication is infection-induced enlargement of the cell because of fluid retention within its walls. This eventually breaks due to the excessive pressure build-up within, thereby giving rise to disintegrated hepatopancreas tubules^83^. Similarly, Bhavan and Geraldine^75^ reported that hypertrophies can be caused by thickening of the basal laminae of the tubules of the infected hepatopancreas. According to Nadella et al.^81^, such occurrences are good indications of the early defense of cells in reaction to the presence of a pathogen.

Invertebrates do not have specific, adaptive immune responses as vertebrates do; therefore, their immune responses depend exclusively on the innate, nonspecific immune system^84^. Hemocyte infiltration is fairly common in the infected hepatopancreas^72^, as hemocytes play a role in the immune responses of animals to pathogenic invasion^79,81^ and other toxicants^72,76^. These processes include phagocytosis, nodulation, and encapsulation^85^. The infiltration of hemocytes in the tubule is likely due to hematopoiesis (i.e., the process of hemocyte production and maturation). These hemocytes are subsequently released into the circulation system to replace damaged or damaged cells for stable cellular function^86^. Microorganism infections were reported to potentially reduce hemocyte counts in crustaceans, but the system was restored once the infection had stopped^87^. Hence, the rapid recovery of hemocytes is important for the rapid eradication of pathogens^88^. Therefore, the ability of crustaceans to produce hemocytes at an optimum rate is important for ensuring survival under stressful conditions. The appearance of healthy hepatopancreatic cells among the severely damaged hepatopancreas tissues is in line with the findings of the study by Sreeram and Menon^89^. According to Boudet et al.^72^, the hepatopancreas can recover and restore its structure during and after stress conditions. Therefore, the results of this study suggested that supplementation of *P. vannamei* PL with the methanolic extract of *M. citrifolia* fruit potentially improved the defense and self-recovery of the hepatopancreas following challenge with *V. parahaemolyticus.*

## Conclusion

This study showed that *M. citrifolia* fruit methanolic extract supplementation in *P. vannamei* PL nutrition effectively improved resistance against *V. parahaemolyticus* during the challenge test. The best dosage of *M. citrifolia* fruit methanolic extract supplementation found in this study was the highest dosage (50 mg/g), which gave survival of up to 26.7% more than the control. The outcome of this study revealed that the enhanced immunity of the shrimp against *V. parahaemolyticus* may be associated with improved hepatopancreas health resulting from extract supplementation. This was shown by improvements in the digestive and antioxidant systems, hepatopancreas cells, and histology. Future studies can therefore test the effects of *M. citrifolia* fruit extract on other aquaculture species and diseases in an attempt to determine the versatility of treatments. Additionally, the potential of other parts of the plant could be the focus of future research to elucidate the biocontrol ability of these plants for aquaculture diseases (Supplementary information).

### Supplementary Information

Supplementary Information 1.

Supplementary Information 2.

Supplementary Information 3.

Supplementary Information 4.

Supplementary Information 5.

Supplementary Information 6.

## Data Availability

The data for this study will be made available upon request from the corresponding author.
